# The Asylums of Italy, Germany, and France

**Published:** 1857-07-01

**Authors:** John T. Arlidge

**Affiliations:** Member of the Royal College of Physicians of London; formerly Resident Medical Officer of St. Luke's Hospital, London


### ART. VI.—THE ASYLUMS OF ITALY, GERMANY, AND FRANCE.

NOTES OF A VISIT MADE IN THE YEAR 1855.

BY JOHN T. ARLIDGE, M.B., A.B. (LOND.),

Member of the Royal College of Physicians of London; formerly Resident Medical Officer of St. Luke's Hospital, London.

DURING a rather lengthened tour on the continent of Europe and in the East, I took the opportunity of visiting those asylums of the different countries through which I passed, that were not too far out of my road, and made notes at the time both of the leading features in their structure, arrangement, and management, which struck my attention, and of the facts which I could gather from the physicians and others superintending them. But as the time and opportunities of observation and inquiry varied much in different cases, the account I can render of some asylums is much more incomplete than that of others. Sometimes I had the advantage of being conducted through an asylum by the medical director or superintendent, whilst at others an assistant medical officer, an interne, or even, on a few occasions, a non-professional *employé* acted as my guide, and was often wanting in much information which I could have wished to have obtained. Another circumstance, affecting, in some instances, the completeness and utility of my narrative was that, from want of time, or some incidents of travel, my inspection was rendered too superficial and hurried to be satisfactory. Lastly, although, in a few cases, I was enabled to obtain some printed returns, containing statistics and other matters of interest, yet, in the majority of these continental institutions, no such reports are procurable, since they are made only in manuscript to Government officials, to the bureaux of the police and the like.

However, it is my duty and pleasure to state that, wherever I applied for admission to an asylum, as an English physician interested in the treatment of the insane, I was readily admitted, and treated with courtesy, indeed, in most cases, with the greatest kindness, and with the readiest will to supply me with all the information I could ask for. The friendly reception which, I may say, everywhere met me, will indeed be always remembered with the liveliest satisfaction and pleasure.

Among other matters, I much wished to acquaint myself with the lunacy laws of the several countries visited, but found it impracticable as a traveller to do so, since they are either not published apart from the general code of laws, or not procurable of any publisher, being communicated only to those specially concerned in their operation, or in some places treated like state secrets. To persons resident for a length of time in any foreign country, a knowledge of the laws regulating asylums, and the management of the person and property of lunatics, might undoubtedly be obtained by a search through their law-codes, and by communications with the directors of asylums, which might supply many useful suggestions for the amendment of our lunacy act, for the more efficient inspection of our asylums, and for securing better provision for our pauper lunatics, especially when recently attacked.

*Asylums of Italy.*—Information concerning the Italian asylums is very scanty; indeed, the very locality of many of them is generally unknown. That they are very much behind both in structure and management, when compared with those of the greater part of the rest of Europe, has been a statement repeated again and again since the time of Esquirol, yet few have inquired for themselves into its validity within a recent period, although nothing is more supposable than that the ameliorations carried out in other countries have also been adopted in Italy. In fact, in several parts of the Italian peninsula improvements in the construction of asylums and in the management of the insane have been carrying on for many years, and the Italians can assume much credit to themselves for the work they have accomplished. They claim, indeed, the merit of taking the initiative in improving the condition of the insane, by employing them in manual operations, by transforming their habitations from prisons into asylums, by planting gardens for their exercise, occupation, and amusement, and, in general, by substituting plans of moral discipline, where practicable, in lieu of mechanical coercion. In these reforms the asylums of Palermo, in Sicily, and of Aversa, near Capua, appear justly to have obtained the chief merit, but the question of priority in their introduction cannot at present be examined.

*The Royal Asylum of Turin.*—The Royal Asylum of Turin was instituted in 1728 by the Confraternity of St. Sudario, who obtained from the king, Victor Amedeus II., the privilege to erect a building for the care and treatment of the insane, by subscriptions raised among themselves, and from the public, and to undertake the management. The site chosen was unfortunate, being at the northern angle of the city, where it is rather low and ill-drained. The first building was very limited; but by subsequent enlargements, between the years 1765 and 1820, was rendered capable of receiving fifty patients. However, its insufficiency for the increasing number of inmates, and for their proper classification and treatment, led to its abandonment, and the erection of the present asylum in 1833, from the designs of the Cavaliere Talucchi. The expenses were borne by contributions from the Government and from private donors, especially from the members of the lay brotherhood of St. Sudario, the founders of the first institution. The patients were transferred to the new building in 1834.

The government is vested in a body of sixteen directors, annually selected by the king from the brethren of St. Sudario, presided over, *ex officio*, by the Vice-President of the Piedmontese Senate. The directors are always eligible for re-election, and consequently remain in office usually for several years. Each director specially assumes to himself the supervision of some particular service, as, for instance, the economical details, the sanitary condition, or the dietary, and for this purpose attends daily at the asylum for his turn of fifteen days, when he is replaced by another. A general meeting is held every Sunday, at which propositions for reforms and improvements are submitted to consideration. The regulations of 1837 require that one physician of eminence in the city shall always be a member of the administrative board.

The paid officers consist of a rector or clerical director, a chaplain, a treasurer, a secretary and assistant, a chief physician, a second physician, a surgeon and two assistants, a dispenser, seven Sisters of Mercy, and inferior officers and servants. All these *employés*, except the clerical director and the surgeon, live in the institution, and the order of duties of the medical officials is so arranged, that one is always present in the house. The internal management will be more satisfactorily considered after the constructional details are gone over.

The chief physician, who has laboured in this asylum for twenty years, is Dr. Bonacossa,—an admirable example of a physician, who has his heart in his occupation, and is constantly engaged in endeavouring to alleviate and improve both the condition of the insane, and the institutions for their reception in his native land. To him I owe my best acknowledgments for his kindness and cordiality, as well as for his readiness in putting into my hands whatever information he could give me.

The present asylum retains the same situation as the one it replaced. It is built on a nearly level spot of ground, surrounded on all sides by public roads, and much overlooked by neighbouring houses, although encompassed by a high wall. In its length it extends from east to west, and consequently its chief fronts are north and south, the former being much exposed to the cold winds which blow frequently with much rigour at Turin from the snowy Alpine range within sight of the city. More than a fourth of the whole superficial area within the walls is occupied by the building itself, and hence little space is left for exercising courts or for out-door employment.

The disadvantageous site and position of the asylum, and also the want of space about it, have been forcibly set forth by M. Bonacossa in several memoirs addressed to the Government and to the Board of Directors. The deficiency of space for out-door employment has, for the last few years, been remedied by an auxiliary institution, started in the country, some few miles from Turin, where a select number of male patients are sent and employed in agricultural operations; a plan which has worked most advantageously, thanks to the enterprising physician-in-chief, and one which he hopes shortly to see extended. This gentleman, however, proceeds further in his views of reform, for he advocates the abandonment of the present building in the town, and the establishment of one in the country in its place. In this recommendation every English superintendent will coincide, especially when acquainted with the defects of the present building.

The asylum is constructed of brick, plastered or stuccoed over. Its ground plan represents two parallel lines of building, connected at the extremities by two others, at right angles to them, and separated into two equal divisions by a central, transverse portion. This central building contains the residences of the officers and the general offices, and serves to separate the male from the female side, and is like the terminal or connecting wings of three stories, the long intermediate portions having only two. The whole edifice is also raised some three feet and upwards above the level of the soil by a vaulted sub-basement, which serves for the stowage of wood and other stores, and beneath the central portion contains the kitchen and associated offices.

From the plan of the building two distinct interior courts are formed, surrounded on all sides by the walls, access to each being provided beneath the connecting end wing, which is built upon an arch. Beneath this arch, on each side, is, moreover, the entrance to the asylum from the garden, or airing courts; a clumsy arrangement, involving the evils of an obscure, heavy, and inconvenient entry, additional stairs, and a dark and otherwise bad staircase.

These internal airing-courts are practically useless; they are too narrow—not above twenty-four feet wide—are always more or less completely in shade, and consequently rather damp; are overlooked from the corridors above, and in general dull and cheerless. They are paved with stone, and slope from the walls on each side towards the centre, for the purpose of carrying off water. The floor of the corridor of the ground-floor is some eight feet above the level of these courts, which are somewhat sunk in order to admit access from beneath the arch communicating between them and the exterior plot of ground.

This general plan of the asylum is doubtless very objectionable and fraught with inconveniences; but since its deficiencies are sufficiently evident, a glance at the most prominent will here suffice. Forming, as each half does, a hollow square, the opposite sides are so approximated that the ventilation and lighting of the wards must be considerably interfered with, and the enclosed courts always in shade and dull; at the same time, every prospect is cut off, and those dwelling on the one side looking inwards to the court can only gaze upon their neighbours opposite through the bars of the windows and corridor.

The central building is devoted to the general offices of the establishment. In the sub-basement are the kitchen and appurtenances, the wash-house and dead-room; on the basement, or ground-floor, are the chapel, dispensary, secretary's office, physician's-room, a billiard and visitors' room, and a dining-hall, or general sitting-room for tranquil patients; on the floor above is a store-room, for the distribution of bread, wine, rice, and other articles of diet, and some living rooms for officers; whilst on the highest, or third floor, are the linen-store, or "lingerie," a work-room for women, and a laundry, constituting a department where the female patients are employed during the greater part of the day.

The asylum is entered from the south side by an open iron-gate, flanked on one side by a porter's lodge, on the other by a small office, which conducts to a portico and arched door-way, opening into a small inner court, formed by the apartments of the central building being arranged as a hollow square, having its rear occupied by the chapel. On each side the entrance-hall, a staircase ascends to the floor above.

The transverse terminal wings, joining the two principal lines of building, are also of three stories, with a sub-basement. The last is pierced at its centre by a wide arch, opening a communication between the interior court and the exercising court outside, and having on each side a staircase, ascending respectively to the anterior and posterior range of building. The ground-floor is taken up by a day-room, a bath-room, and other apartments belonging to the wards on that floor, front and back. The two floors above are occupied by the higher classes of pensioners, and have a row of chambers on each side a narrow corridor, which is consequently badly ventilated and indifferently lighted. To those pensioners who pay from 700 to 1000 francs a year, separate rooms, in some instances a living and a sleeping-room, are assigned, fairly furnished and sufficiently comfortable. Military officers pay only about 320 francs per annum, but rank in the second class of pensioners.

The remainder of the building is devoted to the poor, the annual charge for whom, including clothes, is only about 220 francs. Four-fifths of their cost are paid for by the province to which they belong, a part and even the whole sum, however, being repaid out of the means of the patient, or of his friends, where these allow it, and one-fifth contributed from the funds of the asylum.

The ground-floor on each side is divided into four dormitories, an eating or sitting-room, a few small rooms and a bath-room. The dormitories differ in size. The largest is used for the infirmary, and contains at least twenty-four beds, whilst the rest have only some eight or ten. There is besides a small room adjoining the infirmary, which serves both as a dining and sitting-room, and, like the latter, is furnished with a stove.

As originally constructed, these dormitories had a wide corridor —about eleven feet—on each side, and consequently received light and air indirectly; but one of them, M. Bonacossa, has thrown into the rooms,—a great advantage doubtless, by giving increased space, air, and light, but lessened by the intersection of the rooms by the heavy square columns left. The retained corridor serves for communication; it runs along on the side of the enclosed court, into which it looks. Its outer wall is supported by a series of square columns, the wide spaces between which are filled up by a lattice of stout wooden bars.

There is a bath-room on this ground floor, to the anterior and posterior division, on each side. The baths are of stone, shallow, narrow, and furnished with stout wooden covers, capable of being firmly fixed down. They are four or five in number, and disposed in a radiating manner around a central column, through which they receive the water. The douche-pipes are also suspended from this column over the baths. Bathing is not resorted to for the purpose of cleanliness, but only where indicated medically, or morally—*e. g.*, as a means of repression. Prolonged baths are sometimes used with advantage to produce sleep and allay excitement. A small stream of water is usually allowed to trickle over the head during the whole time, and occasionally liniments, or ice, to the head, are employed. Cold baths are very rarely resorted to.

The first floor, or second story, consists of a wide central corridor (20–24 feet), having a series of single rooms on each side. It is lighted indirectly through the rooms, and directly by a row of semicircular windows, on either side, some distance above the rooms. This line of windows, seen from without, gives the impression of a third story, or attic; for they are placed above the level of the parapet, and so set back from the outer wall that they pierce the inner one (partitioning the single rooms from the central gallery), which is continuous upwards to the ceiling. This mode of construction gives great elevation to the exercising gallery, but its effect is dreary, and no views outside can be obtained, except through the windows of the sleeping-rooms. Moreover, the gallery being extended to the extremity of the building, through the intersecting terminal wings, which have two stories in the elevation of this corridor, its height is abruptly and seriously diminished, to about ten feet, by an arch thrown across it to support the superjacent rooms. This low pitched portion, nearly a fifth of the entire length, is badly ventilated and lighted, since there is neither an end window, nor rooms laterally through which it can receive light and air. It follows that this form of central corridor is very unsatisfactory, and, except for the purpose of in-door exercise, has no one feature to recommend it. The architect has undoubtedly perceived the disadvantages attending a corridor with rooms opening into it on each side, and to obviate them by procuring light and air directly from without, instead of second-hand through the rooms, resorted to the curious expedient of elevating it much above them. At the same time he fell into a great mistake by continuing it through the intersecting terminal wings, and thereby produced a most clumsy interior, cut down to one-half its height by a bare heavy wall into a sort of communicating tunnel.

The walls of this gallery are plastered, and are coloured to the height of a chair-rail of a different colour, the rest being whitewashed. The whole seemed kept in very good order.

The single rooms on the first floor are about 11 feet by 9 feet, and 11 feet high, with slightly arched ceilings, and the walls plastered and whitewashed. Some rooms are slightly larger than others. Each has a window defended by ornamental iron-work outside, nearly 3 feet wide, and 4½ feet high, and placed some 4 feet above the floor.

Each ward or gallery has its water-closet, which is of the rudest construction, and, to English senses, dirty and disgusting. It is, however, but just to say, that M. Bonacossa recognises their defects, and is anxious to have them replaced by something more sweet and decent, but he has to encounter the "*laissez-faire*" system, which suggests that they have served and may still serve their purpose, and are after a pure native model. The closet, as it existed, was really a small room with a stone floor, having one hole in it for solid excrementitious matters, and another, with a groove leading into it, for the liquid contributions; but the latter especially seemed frequently to miss their way, if we might judge from the wet condition of the floor and the odour arising from it.

Besides serving as a place of exercise, the wide corridor constitutes also the eating and sitting room for the inmates, wooden tables being placed here and there for taking the meals on, with benches and chairs.

The stairs, ascending from one floor to another, are of stone, and although their course is not spiral or winding, yet they are disposed around a central space or well, and, consequently, to render them safe, are shut off from it by upright iron bars, giving them, as in those of the Hanwell Asylum, a cage-like and very objectionable appearance. Moreover, they are but indifferently lighted by a skylight in the roof above.

The floors of all the dormitories, single and sitting or eating rooms, are laid with square tiles; those of the gallery with stone. In the floor of the bath-rooms alone is wood used.

The ground on which the asylum stands is so much occupied by it, that but a narrow strip is left before and behind it: the principal space for exercising-courts and gardens is found at each end. All the available space is made use of; one portion on each side is set apart for the refractory, and is without trees or shrubs; the remainder is divided into two gardens, laid out with paths, turf, trees, shrubs, and flowers. A larger garden is also attached to the building on the south aspect, placed on the other side of the public road which bounds that side of it, and is reached by a tunnel passing beneath. This plot of ground was the garden of the ancient asylum. As many male patients as practicable are employed in keeping the gardens in order, but from the limited space few can obtain the advantage of out-door employment, an evil much lessened, for the last two or three years, by the acquisition of a monastery and its grounds, as an auxiliary establishment, where above 100 patients reside, and are engaged in cultivating the soil and other work.

Respecting the internal fittings, a few words will suffice. Simple iron-bedsteads, of a common pattern, are used generally throughout the asylum. For epileptics they are furnished with stuffed sides, to prevent the patients falling out during a paroxysm, and to obviate injuries. Clean patients have a flock-bed and a straw-paillasse, with sheets and a thick coverlet. For dirty patients, the bedsteads have the sides raised so that they may contain an iron or wooden tray, in which the straw upon which they lie is placed. The straw is not enclosed within a case or bag, but placed loosely in the box, and a sheet laid upon it, for Dr. Bonacossa thinks that bags prevent the urine readily running off, and therefore make the state of the bed worse. The urine drains off through a hole in the bottom of the tray, and is collected in a vessel beneath.

The drinking utensils are of pewter. The dress for the poor consists in summer of cotton, in winter of woollen cloth. No special provision is made for warming or ventilation; the cold state of some parts of the building is deplored by M. Bonacossa, who attributes to it the too frequent prevalence of disease and an increased mortality.

The internal government and management of the asylum seem very much influenced by the directors; the medical treatment being regarded as the special province of the physician. He, however, directs his attention to all the details, and is ever active in urging reforms in all matters affecting the well-being of the afflicted inmates. Yet he seems too much under the control of the directors to effect the good his experience suggests to him to be done. This indeed he himself feels, and has boldly petitioned the state authorities to place all the asylums of the country under the superintendence of a medical director. Whilst the chief physician has, in some sort, a general supervision of the institution, he shares equally with his colleague in medical duties; the one charging himself with the male, the other with the female, side of the house. They each make three visits to the patients in the course of the day,—the first early in the morning, the second at noon, and the third in the evening. The chief has also to prepare a weekly return to the directors of every occurrence in the asylum, of the patients admitted and discharged, of the form of insanity they suffer, and to compile the statistics every three or six months. The surgeon, who is non-resident, visits daily and conducts the autopsies. The two assistant-surgeons follow the superior medical officers in their visits, perform the minor operations of surgery, supervise the administration of medicines and the diet, keep registers of the cases, &c.

Seven Sisters of Charity are engaged in the service of the asylum; three of whom superintend the laundry and clothing department, whilst the rest act as the chief nurses on the female side, where they are assisted by ten under-nurses. On the male side there are, a head and fifteen ordinary attendants, and a barber. Besides this staff, there is a nightwatch on each side. The rector has the moral control of the asylum, and with the resident chaplain conducts the religious services—viz., prayers every evening, a lecture once a week, and the sacrament at certain times to those of the patients in a condition to receive it.

In the kitchen are a head cook and two assistants. The steward, who is also the chaplain, is charged with the inspection and distribution of all the stores, and reports on the dietary, &c., to the director who presides over the economy of the house. Additional servants are, a man to keep in order the clothing of the male patients, and an under-steward or butler, who gives out the portions of food, wine, &c., for the inmates.

There are three meals per day; the first between 7 and 8 A.M., the second between 11 and 12, and the third at 6 P.M. The morning meal consists of soup and 3 oz. of bread; the mid-day meal of soup, 12 oz. of bread, and a portion of meat four times a week, with or without vegetables; on other days, the soup is made with rice or macaroni, or eaten with vegetables. The evening fare is of soup again, or of salad, with 6 oz. of bread. Besides this allowance of common bread, a little of the Turin "pipe-bread" is given in addition, and a quarter of a pint of wine mixed with water, is allowed at the principal meal. The private patients or pensioners have extra portions and a greater variety of food, regulated by their amount of payment. These take their meals separately in their own rooms, whilst the other inmates eat together in their several wards. As many as 130 sit down at meals in the division of the tranquil patients; knives are not in general use, but spoons and forks, the former of white metal for the pensioners, and of wood for the pauper inmates.

Little employment is obtainable in this asylum, particularly for the male patients. A few, as said before, are busied in the grounds, others in the wards, but there are no workshops. A

library and reading-room are provided, and also a billiard-room for the pensioners. The women are engaged in needlework, in the laundry, and in assisting in the work of the wards.

No suicide had happened for fifteen years.

Restraint is still resorted to in violent cases; the hands being fastened together by leathern handcuffs, or attached to the waist by a belt, or a camisole is put on, and occasionally a patient is confined in his bed. Coercion, however, is limited in the greatest degree possible. Seclusion is frequently practised, sometimes in a darkened room.

The number of patients in the establishment, at the time of my visit, was 533—of these 217 were women and 316 men; among these there were 19 epileptics—12 males and 7 females, and 13 paralytics.

In 1846, as many as 320 patients were admitted in the course of the year, of whom 14 men and 11 women were epileptic, and 18 men and 8 women paralytic.

From the faulty construction of the building, the proper classification of the inmates is impracticable. They are generally divided into the refractory and the tranquil or orderly and clean, but the epileptics, the dirty demented, and paralytic are mingled together. The deficiencies of this division are distinctly recognised by M. Bonacossa, who would add to the usual sections, one for the recently admitted and another for criminal lunatics.

In 1837, a code of regulations agreed upon required the insane to be grouped under six classes—viz., the convalescent, the maniacal, the epileptics, the quiet incurable and imbecile, monomaniacs and melancholics, and those subject to paroxysmal mania; an elaborate division, but impracticable in the existing building.

The so-called moral treatment appears carried out in most points so far as circumstances will admit; excitement is quieted by removal from its cause or by seclusion; the mind is diverted and occupied by exercise in the courts and gardens, and at times by walks outside the asylum grounds by employment in the building or at its branch, by dancing, music, plays, puppets, reading, billiards, &c.; and its moral powers exercised by the religious services and instruction daily carried on.

The medical treatment is necessarily varied by the special features of the case, but speaking of its general type, it may be called depressing. Bleeding, either general or local, according to circumstances, is practised in recent cases, chiefly of mania; also full doses of emetics, digitalis, cherry-laurel water, hyoscyamus, and belladonna, are in constant use; but opium is very seldom administered. Purgatives are given in melancholia, but not the very drastic sort, and repeated several times; cauteries and setons are used in dementia. For epilepsy, zinc, indigo, cherrylaurel water, copper, nux vomica, and strychnine, have been tried with little or no success.

Prolonged warm baths are found very useful in many recent cases, and are sometimes medicated for use. The douche is occasionally resorted to for the purpose of repression, or, in some measure, as a punishment. Shower baths are unknown.

On a review of the preceding account, it must be admitted that much credit is due to the management of the Turin Asylum. The defects in its construction are such as necessarily entail many inconveniences and evils, and unfortunately, are almost completely irremediable. The situation within the circuit of a large city, the manner in which it may be overlooked from neighbouring houses, the small space about it, the double line of building with closed ends forming a hollow square, the disposition of bed-rooms on each side a gallery, and the want of workshops and of means of warming the building in winter, are among the conditions almost or quite past remedy in the present institution, and which will always render it unfit for an asylum, more especially so for the treatment of recent cases. It is to be hoped, therefore, that M. Bonacossa's proposition to set aside the present structure, and to erect a new asylum in its stead in the country, will ere long be attended to.

It is gratifying to see the humane principles of treatment of the insane so cordially accepted in the Turin Asylum, and to know that most of the defects of the present building and of its internal management are admitted. Although personal restraint is still regarded as necessary in some cases, yet the constant endeavour is to reduce it to its minimum. The dietary to an English stomach would be considered very meagre and insufficient for health, yet it is actually somewhat superior to that obtainable by the majority of the poor in Piedmont. Nevertheless, both from *à priori* considerations regarding the depressing effects of mental disease, and the change in the mode of life of most of the inmates, the deprivation of the invigorating air of the open country, the confinement and inactive dreariness of an asylum, and from experience of the indifferent sanitary state of the establishment, the prevalence of diarrhœa, dysentery, and scurvy, M. Bonacossa very justly demands an improvement in the quality and quantity of the diet. On this subject, he further points out that the frequently fatal maladies named are especially rife among the poor who have the bare allowances prescribed, and, on the other hand, are much scarcer among the private patients whose payments secure them a more varied and substantial regimen. The scanty charge of 220 francs (9*l*. of English money) for the destitute cases is found insufficient to allow a more generous diet, and to provide superior clothing to that which is at present supplied,—a reform much called for; it is therefore necessary that this sum should be augmented.

The thanks of those interested in the welfare of the insane are, moreover, due to M. Bonacossa for his persevering endeavours to provide employment, particularly for the male patients, by attaching land to the asylum for cultivation, and by the erection of workshops for the exercise of various trades. Another change he advocates is, that the private patients should dine at a common table instead of singly; but he does not approve of the two sexes being brought into contact with one another. We are glad, also, to quote this able physician's opinion against large asylums, which he states produce, *cæteris paribus*, much less satisfactory results than smaller ones, and he complains of the present size of his own, which is, indeed, rendered more objectionable by overcrowding. Lastly, the present number of attendants for the patients is found inadequate, being but one to fifteen; and M. Bonacossa would desire to have one to ten, and that their stipend should be greater, to secure more activity and interest in their duties.

M. Bonacossa is the author of several works on the Statistics and on the Pathology of Insanity. He understands English, and is diligent in ascertaining the prevalent doctrines respecting the management and treatment of the insane, both in this country and elsewhere. In 1838, he made a tour of inspection to several of the asylums of France, Holland, Belgium, Lombardy, and of this country, and published his notes in a small book, entitled "Sullo Stato de' Mentecatti e degli Ospedali in varii Paesi dell' Europa."—Turin, 1840. In 1837, he published the Statistics of the Turin Asylum, between 1831 and 1836, under the title of "Saggio di Statistica," prefacing the work by remarks on the history and government of that institution, on the classification and treatment pursued, &c. On the proposition of a new code of laws for the regulation of lunatics and lunatic asylums, he wrote a brief memoir containing comments and criticisms, suggesting amendments; but his last and most ambitious work is a treatise on mental pathology, entitled "Elementi Teorico-Pratici di Patologia Mentale," printed in 1851, which we can recommend to the careful study of our readers as rich in original thought, and as conveying the results of some twenty years' reflection and experience enjoyed by the author in the Turin Royal Asylum.

From these additional sources of information, not a few interesting particulars can be advantageously collected and appended to our own notes on the asylum under consideration.

The mortality in this hospital appears very high, and is attributed by M. Bonacossa to its defective hygienic conditions; to its position, its exposure to the cold north-west winds, and to the insufficient diet. At the same time, he would explain it in part, by the extent of the movements of its population in the course of a year—from 250 to 260 having of late been admitted yearly—and by reference to the state of health in which many patients are sent to the asylum, especially those from rural districts and from long distances, as from the province of Nice. Whatever explanations of this sort may be advanced, there is certainly a vast amount of sickness in this asylum; for, including relapses, five per cent. more cases of disease (incidental with reference to the insanity) occur than the entire number of inmates. But to revert to the ratio of deaths:—the statistical tables show, that between January 1st, 1831, and December 31st, 1836, 650 men, and 416 women, in all, 1066, were admitted; that 161 men, and 109 women, in all, 270, were cured; and that 188 men, and 140 women, in all, 328, died. Thus with reference to the number of admissions, 1 in 4 were cured, and 1 in $3\frac{1}{4}$ died; the ratio of deaths exceeding that of the cures.

According to another table, there were:—

| | Admissions. | Deaths in the year. | Ratio. |
|---|---|---|---|
| In 1830 | 165 | 23 | $1:7\frac{4}{23}$ |
| 1831 | 149 | 20 | $1:7\frac{9}{20}$ |
| 1837 | 207 | 49 | $1:4\frac{11}{49}$ |
| 1843 | 256 | 52 | $1:4\frac{38}{55}$ |
| 1845 | 267 | 61 | $1:4\frac{23}{61}$ |

This increasing ratio of deaths with that of the admissions has been observed to be a necessary law by many asylum physicians, and if applied to the statistics of the Turin Hospital, the rate of mortality for the last two years cited should have been greater, had not some amelioration in its sanitary condition have been effected.

To show the curability of insanity when submitted to treatment soon after its outbreak, the following table is presented, which, in the facts it conveys, coincides with the results of experience of other physicians.

During the first three months after the invasion of the malady, 3 in 10 are cured.

| | | |
|---|---|---|
| second ditto | 3 in 12 | ” |
| third ditto | 3 in 13 | ” |
| fourth ditto | 3 in 20 | ” |
| After the lapse of a year | 3 in 33 | ” |

The teachings of experience seem, however, equally ignored by the people of Piedmont as by those of England, for M. Bonacossa has to regret that the majority of cases admitted are of more than a year's duration, and three-fifths of the whole without prospect of cure. Still, in Piedmont, something will no doubt be done by the central government to provide for the immediate treatment of recent cases, when its importance, as well to the state as to the interests of the afflicted patients, is placed in its proper light; whilst in our own much-lauded land of liberty, obtuse-headed magistrates will be suffered, apparently *in perpetuo*, to ignore the deductions of experience, and to persist in repeatedly enlarging asylums already greatly overgrown, which are suitable only to give safe custody to the insane, and, by depriving them of all chances of sufficient treatment, are in operation no other than manufactories of chronic insanity.

The much greater proportion of males admitted to females is remarkable; it represents three-fifths of the former to two-fifths of the latter,—the reverse of the ratio in England, France, and Belgium. Among the males, agricultural labourers rank first in the tables of the relative occurrence of insanity among different occupations; after these come priests and military men. Among other social conditions no striking diversity exists, except in the case of shoemakers, who exceed any of the rest in relative number. Of the 650 men admitted between 1831 and 1836, 233 were labourers. A similar predominance obtains among women of the same class; since, of 416 admitted, 195 were labourers, and 58 were similar in social position, viz., servants. This prevalence of the disease among the labouring classes is doubtless attributable, in a great measure, to their wretched mode of life, and their half-nourished condition, particularly in the mountainous districts, and to their ignorance and superstition.

With respect to age, the greatest number of cases occur between the thirty-fifth and fortieth year.

The unmarried, as a rule, suffer more frequently than the married; an exception, however, obtains in Piedmont, in the case of the females, of whom more married are there admitted. Hereditary predisposition was traceable in one of every four or five cases admitted. Next to this, abuse of wine and spirits follows as the most frequent cause, especially among the men; also not a few cases are attributed to pellagra, although this malady is not nearly so prevalent in Piedmont as in Lombardy. An unusually large number, in comparison with other countries, suffer from superstitious monomania, from demonomania, and with aversion to food. Epilepsy coexisted with insanity in 30 out of 557 males, and in not more than 5 of 388 females admitted. General paralysis is infrequent in Piedmont, although mania with exalted notions is commonly met with. M. Bonacossa narrates the surprise of a French physician from Charenton on recognising this circumstance in the course of a visit to the Turin asylum, he having been accustomed to look on this particular form of delusion as almost necessarily connected with paralysis.

The most prevalent forms of insanity are mania and melancholia; the former very often intermittent in type, and the latter attended with excess of religious emotion, and mostly with fear of eternal punishment, and seen more frequently among women than men. A tendency to suicide exhibits itself in a fourth of the cases on their first entrance, and aversion to food is a tolerably frequent complication of melancholia. Dementia holds the third rank in relative frequency, and after that, in women, erotomania; monomania with extravagant notions follows.

Taking a period of eight years, and including in the calculated number of cases those remaining in the institution at the commencement of that period, M. Bonacossa finds the proportion of cures to have been 28 per cent.; that of discharges under all circumstances 45 per cent.; that of deaths between 35 and 36 per cent. The ratio of relapses is from 11 to 12 per cent. The average time spent in the asylum by the cured was 220 days; by those who died, 306 days; and by the inmates generally, under all circumstances, 265 days.

On looking over the table of the causes of death among the population of the asylum between January 1st, 1831, and December 31st, 1836, one is much struck with the high mortality resulting from disease of the abdominal viscera, especially of the gastro-intestinal canal. In seeking an explanation of this fact, we are at once led to assign much importance to the dietary in the first place, and to the bleak and rather damp position of the asylum in the second. Some influence must also be assigned to the vitiated air from imperfect ventilation and drainage, and to the cold of winter, inadequately mitigated in only a few apartments by stoves. The table is sufficiently interesting to extract at length.

| Causes of Death. | | Proportion of Deaths relative to the various Forms of Disease. |
|---|---|---|
| Of the Brain and Spinal Cord | chronic myelitis | 1 in 52 |
| | meningo-arachnitis | 1 — 8 |
| | acute encephalitis | 1 — 130 |
| | encephalitis and meningitis | 1 — 10 |
| Of the Vessels | chronic phlebitis | 1 — 86 |
| | arteritis and carditis | 1 — 37 |
| Of the Thoracic Viscera. | phthisis pulmonalis | 1 — 18 |
| | chronic pulmonic disease | 1 — 18 |
| | acute pleuro-pneumonia | 1 — 86 |
| Of the Abdominal Viscera | chronic gastro-hepatitis | 1 — 37 |
| | acute hepatitis | 1 — 160 |
| | chronic gastro-enteritis | 1 — 13 |
| | chronic cystitis | 1 — 86 |
| | acute metritis | 1 — 260 |
| | chronic metritis | 1 — 130 |

| Causes of Death. | | Proportion of Deaths relative to the various Forms of Disease. |
|---|---|---|
| Fluxes | dysentery | 1 in 32 |
| | diarrhœa | 1 — 13 |
| Cachexies | ascites | 1 — 52 |
| | hydrothorax and hydropericardium together | 1 — 18 |
| | hydrothorax | 1 — 37 |
| | hydrocephalus with meningitis | 1 — 10 |
| | scurvy | 1 — 65 |
| | tabes mesenterica | 1 — 65 |
| Neuroses—Apoplexies | serous | 1 — 21 |
| | sanguineous | 1 — 18 |
| External and Surgical Diseases | | 1 — 50 |

In the history of the Turin asylum the year 1837 holds a prominent place on account of the introduction, at that period, of extensive modifications in the internal management and modes of treatment; the summary of which, as detailed, furnishes an insight into the state of this institution prior to that time. As enumerated by Dr. Bonacossa, these reforms were: 1. Daily distribution of the allowances to the poor instead of only three times a week; 2. General adoption of iron bedsteads, and abolition of the old wooden ones with their apparatus of rings, chains, &c.; 3. Separation of the pensioners from the paupers; 4. New and more convenient disposition of the water-closets; 5. Construction of a commodious bath-room in every section; 6. Repairing of the floor with tiles instead of stones; 7. Altering the chamber doors to open outwards instead of into the rooms; 8. Institution of a reading-room; 9. Cultivation of the garden of the ancient hospital by the labour of the patients; 10. Two assistant-surgeons appointed in lieu of a phlebotomist; 11. One physician specially attached to the male, the other to the female division; 12. Opening of the dispensing department (pharmacy) to the public for the profit of the asylum funds. Besides these, adds the writer, many other improvements would have been brought about, had the new regulations of 1837 been acted upon with vigour.

This Royal Asylum at Turin was lately, except a small one at Alessandria, the only institution which existed in the whole of Piedmont (Genoa being a distinct province) for the reception of insane persons; and, besides doing duty for the population of this large territory, the patients belonging to the province of Nice were also sent to it. The utter insufficiency of this one institution for the claims upon it forms the subject of a paper addressed to the central government by Dr. Bonacossa, who forcibly points out the many and various evils resulting from such a state of things.

Within the last few years the regulations for the education of medical men in Sardinia require students to attend, in their fifth year of study, the clinical visits at the asylum, and a course of lectures on mental disease. It is much to be desired that this excellent and most necessary regulation should be enforced in England for all medical students; for the prevailing ignorance respecting the nature and treatment of insanity is truly deplorable.

*The Asylum of Genoa.*—There is a large modern asylum at Genoa at one end of the city, within the walls. It was instituted in 1834, and intended to accommodate 350 patients. It is built of stone on the radiating or panoptic plan, and has a few acres of land surrounding it, laid out in gardens and courts. It is unfortunately overlooked at some parts—particularly the wings on each side the garden, through which the central building is approached, by neighbouring houses. It is situated in a valley, but the drainage appeared good, and the soil dry. The several wings are similar; they are each of three stories, surmounted by an attic running the entire length. The central building, from which the six wings radiate, is circular, of considerable circumference and five stories in height, besides the "lingerie" immediately under the roof.

At the time of my visit there were 423 inmates under treatment; some of whom were pensioners or private patients, paying more or less liberally for their maintenance; and the rest indigent, supported at the expense of the commune to which they belonged, less the sum obtainable from their relatives in part payment.

Each floor in the wings is devoted to patients, except the terminal portions of the two at the approach from the entrance gate, which serve for the residence of the physician, the clerical director, the assistant-physician, and some other superior officers. The general arrangement is that of a central corridor, terminated at one extremity by a window, about seven feet in width, and having rooms (mostly single) opening on each side. However, its continuity as a corridor is broken at the centre of each wing, on the first and second floors, by a room extending the entire width of the wing transversely, which serves as a dining and day or sitting-room.

The single rooms, opening into the central gallery, are commodious,—about 13 feet by 11 feet, and 11 feet high, with a slightly vaulted ceiling. Each has a large window, about three feet from the ground, made to open inwards like a French casement, defended externally by an iron-framework, consisting of perpendicular iron bars, crossed by others obliquely in such a manner as to form an ornamental pattern, less offensive to the sight than the old cross-bars of this country, yet at the best giving a prison-like aspect to the chambers. The panes of glass in the casements are of good size in all the rooms occupied by tranquil patients; but in those for the violent the glass is replaced by wood, except at the upper part, where the only space for lighting was about fourteen inches by twelve. The same style of external iron barricade was attached to the windows throughout the building.

On the first floor of one wing, on each side, set apart for pensioners, tranquil or convalescent, the doors of the rooms have been removed; and are replaced by curtains, which can be drawn across the open doorway, so as to give the necessary privacy. This plan increased the cheerfulness of the ward, which it, in some degree, converted into a dormitory, and must likewise have greatly improved the ventilation both by night and day, the curtains by their mode of hanging and their thinness opposing very little obstacle to the entrance and exit of air, even when drawn, which they frequently are not. Elsewhere, except to the rooms for violent cases, the doors on each side are circular headed, and, instead of being solid throughout, had their upper half filled up with a framework of wood of upright bars only. The circular top to the doors, and the employment of upright bars only, were intended to improve the appearance by obviating the usual prison-bar model; the whole contrivance being, however, called for to secure the lighting and ventilation of the galleries, which, except by the terminal window, were otherwise unprovided for. This indirect and imperfect supply of light gives a dull aspect to the corridors; but this Dr. Verdona (the chief physician) remarked is rather an advantage in Italy, where the light in summer is too intense to be agreeable or desirable for patients. For this reason also it is to be presumed that either a curtain was fixed across the end window of the gallery, or that Venetian shutters were placed outside it; but, whatever the motive, this only general look-out into the surrounding garden, or country, was in nearly or quite every ward cut off; the only other opportunity of the patients seeing the exterior of their habitation being from the windows of their rooms.

The casements of the patients' rooms seemed under their control, to open or shut at their pleasure, there being no special fastening to prevent it. However, the danger of a leap or a fall from them is removed by the iron guard outside. The floors were everywhere of tile or stone, and the walls whitewashed, except for about four feet from the floor, where they were coloured in imitation of granite, or of some other ornamental stone. At some parts the walls were painted in distemper with trees, flowers, &c., according to a taste very prevalent in Genoa. The doors entering the corridors are of wood, and solid. Each corridor is entered from the central building, and has no communication with any other, except by the common stair outside it.

Besides single rooms, most of the wards possessed a dormitory, capable of accommodating some ten patients. In the refractory quarter the doors of the single rooms are solid. A few of them were lined with iron-plates, for the purpose of strength and security, and had a small window for light placed high up out of the patient's reach. There was no padded room in the asylum. Dr. Verdona had constructed one for experiment, but was dissatisfied with it, and gave it up.

The staircases, which are constructed in the central building, are winding; the one leading to the fourth story is, moreover, badly lighted and inconvenient. The fourth story, or third floor, differs in arrangement from the wards below. It is divided into a number of dormitories by transverse partitions, and being within the span of the roof, the ceiling, for part of the way on each side, is shelving. The rooms do not occupy the entire span of the roof, since, in order to gain sufficient elevation, even for the beds, the walls are brought forward from the junction of the roof with the outer walls, four feet or more on each side; hence a sort of horizontal shaft extends the entire length of this floor, on either side, which was turned to the purpose of ventilation. To attain this purpose, small holes were opened from the rooms into this shaft, originally of about three inches square, placed just above the floor. The present medical director has improved upon this, by bringing air from the exterior, instead of from this closed shaft, by extending a square wooden tube across the intervening passage, through the external wall. The tube, about eighteen inches by nine inches, has a septum across it of coarse wire-gauze, intended as a regulator of the current of air admitted, and, with this view, capable of being raised or lowered by a string passing over a pulley, and worked from the dormitory.

But, whatever schemes be resorted to, with a view to improvement, this floor will ever remain an unfit habitation for patients. Situated immediately beneath the roof, the heat of the rooms in summer must always be almost intolerable and unhealthy, whilst the windows, being placed high up in the slope of the roof, deny to the inmates all view, except of the sky, and have a most objectionable appearance, aggravated by their smallness. In fine weather the sun must shine through them with oppressive intensity, and in foul weather they will, like all sky-lights, be subject to many inconveniences.

The dormitories, which open one into another, are under eleven feet in height at the highest part, and contain from six to eight beds each. There was also on this floor a small room, serving as a day and dining room for a portion of the patients. These last were mostly chronic demented cases, but few were habitually dirty in their habits, much care being used to prevent this. The water-closets here, as elsewhere, smelt badly, and were in bad order.

The small rooms, which on the floors inhabited by the pensioners serve only for one patient, are in the other wards occupied by paupers, frequently made to serve for two, an arrangement which cannot be too much condemned, for a variety of reasons not necessary to detail to our readers.

The wards on the ground floor set apart for refractory patients and for epileptics, are deficient in light, gloomy, and not so clean as desirable. Some rooms on this floor were converted into workshops for tailors, shoemakers, carpenters, &c.

The central circular building consists of five stories, the highest of which is appropriated to the linen-room (*lingerie*), and is very well and orderly kept, under the superintendence of a Sister of Charity. The floor beneath is divided into a number of rooms for pensioners, and also contains a billiard-room. The ground floor is occupied by the entrance hall, by a room for receiving visitors to patients, an office for the physician, one for the steward, by the kitchen, &c. Above are the chapel, a common room for general purposes, a store-room, &c.

From the central hall on the ground floor, a door opens on either side, which admits into a smaller hall from which the doors of the several wings on that side open, and give access to the stairs ascending to the various floors above. An inscription appears over the principal entrance of the asylum stating its purpose, in which the insane are oddly enough called "Helleborosi."

From the plan of the asylum, the airing-courts have a triangular figure; two sides being formed by the contiguous diverging radii of the building, and the third or base by a high wall. In two courts an enclosed, partially glazed, wide corridor extends two-thirds the length of the base, intended as a place of exercise in bad weather, and furnished with stone benches; its utility, however, is very limited, and it will probably be removed before long. The airing courts are planted with trees, and most of them also laid out as gardens, with shrubs and flowers. The space entered from the lodge, in approaching the asylum, is larger than the rest, but being overlooked by neighbouring houses, is of little advantage to the patients.

No special contrivance for heating and ventilating the building exists; indeed, the few stoves placed here and there cannot sufficiently warm it in winter; and this season at Geneva is not wanting in severity, especially when, as frequently happens, the northerly winds blow strongly and bitterly over the city.

Respecting the internal fittings and arrangements, there is little to note. Iron bedsteads are generally adopted, made as usual, of stout iron rod, with a head and a foot-piece, and low sides. These sides serve only to contain the thick paillasse, except in the case of bedsteads for epileptics and some paralytics, in which they are much higher, moving up and down on hinges, to serve as a defence against the patients falling out during a fit. The elevated sides, like the head and foot-pieces, are formed by an upper horizontal rod, from which vertical rods descend to the framework of the bedstead. Where patients have thrown themselves about much, and exposed themselves to injury from the enclosing sides and ends, these have been covered or padded, in order to obviate it: with these precautions no accident had occurred.

The bedding consists of a paillasse, a flock bed, sheets, a woollen coverlet, and mostly a coloured cotton counterpane. This provision is made for clean patients: for the dirty, an iron box, fitted to the framework of the bedstead, is filled with loose straw, and a sheet laid upon the top, on the same plan as at Turin: a strong coarse woollen coverlet is placed over the patient. None of the patients were, as before stated, constantly dirty in their habits, but these straw troughs were assigned to such as were prone to be so. Lastly, for destructive and violent cases, some wooden cribs or trough-bedsteads were provided, furnished with iron rings for fastening the straps used as coercive means in certain cases.

Mechanical coercion is applied by the camisole, by the waist-belt, and by leathern hand-cuffs. It is used in violent cases from the apprehension of mischief and injury to others; also for destructive and for suicidal patients; for the last, especially at night. Many of those under restraint by the camisole are also kept in bed, attached to it by belts fastened to the arms, legs, or waist. This mode of management Dr. Verdona prefers to seclusion, believing it much more beneficial to the patient. He would, at the same time, always endeavour to lessen its application and diminish its severity as much as possible, and continue it no longer than absolutely required. The total abolition of mechanical restraint, the use of knives at meals, the mixing together of the two sexes, whether for amusement or religious worship, are, in his opinion and phrase, mere *tours de force*—or as translated—mere knavish tricks to dazzle the eyes and astonish the public.

There is no provision of lavatories for personal cleanliness: in fact, I could not find out that in this, or indeed, in almost any continental asylum, it was deemed at all necessary that patients should habitually wash themselves. The idea of an English wash-hand basin has yet to be realized, and even its accessories, soap and towels, rarely find a place in the *toilettes* of foreign asylums. It seems to be thought enough, *i. e.*, where any such provision is thought of at all, to fix here and there in the building a small vessel of water, holding a gallon, or somewhat more of water, furnished with a tap like that of a tea-urn, and called a "robinet." The effectiveness of a washing from such an apparatus may be conceived; the wetting of the hands and the bespattering of the face with an ounce or two of water, making the patient clean every whit in the estimation of himself and fellows. This arrangement is nevertheless a shade better than the old German model plan of ablution, *viz.*, a glass of water, used *primo loco*, to wash the mouth; *secundo*, to smear over the face; and *tertio*, to damp the fingers, to be dried at the pleasure of the performer with his handkerchief or the tail of his shirt.

However, it is due to the directors of the Genoa Asylum to state that "robinets" were there more numerous than in some other similar institutions; for besides one in each ward, the water-closets generally were furnished also with one; a provision highly commendable when the habits of the people in such conveniencies are considered. It is also right to state that the present medical director recognises the want of more attention to personal cleanliness, and proposes to employ general baths to attain this desideratum.

Knives are not allowed to any patients, but only forks of white metal, and spoons. The denial of knives in this, as in many other continental asylums, will be less felt as a disadvantage than it would be in English institutions, since soup forms the staple article of diet, and when meat constitutes a part, it is usually so much cooked, that a spoon and fork may readily divide it.

The pensioners have each a single room, which is more or less furnished, according to the class they belong to, and the amount of their payment. The furniture consists mainly of a couple of chairs, a night table, a common table, a looking.glass, occasionally drawers for clothes, the bedstead, &c. Books are allowed to many of them, and a billiard table is provided for their amusement. They also engage themselves with drawing, painting, &c., and are permitted, within certain limits, to ornament their rooms, and to amuse themselves with music. The internal economy of the establishment is conducted, under the superintendence of the chief physician and his assistant, by a steward and butler, or pantry-man, and a staff of Sisters of Charity, acting as upper attendants over a number of subordinates, not *réligieuses*. A night watch is kept by two attendants, who sit up all night, and perambulate the building, one on each side.

Three meals are served daily; a breakfast of soup made with vegetables, *legumes*, &c., but not with meat, and a portion of bread; a mid-day dinner of soup made with meat, vegetables, and bread; and a supper, again of soup, not of meat, but of rice or *legumes*, with an allowance of bread. The dietary is superior to that at Turin, and hence diarrhœa and scorbutic affections are less prevalent and fatal.

A large number of the inmates is employed; the men in cultivating and keeping the gardens and exercising courts, in assisting in the wards, in various mechanical trades, and in artistic work; the women in needlework, in repairing and making articles of dress, in washing, in assisting in the *lingerie*, and wards, &c.

A few patients are allowed out of the asylum, in company with attendants, to make purchases in the town, to bring home articles for use, &c.

Among the indigent patients, probably from the insufficiency of the monetary allowance for their maintenance, many were ragged and disorderly in dress, and neglected in person, some without shoes, and many without covering to the head.

Baths are used where medically indicated, and sometimes as a means of repression. For the latter purpose, the douche is more frequently employed where there is violence and excitement to be overcome, or where food is obstinately refused. The œsophageal sound is avoided wherever possible; the use of one spoon to open the mouth and of another to administer the food frequently succeeds. A good plan, which I saw put into operation in the case of a woman who believed evils of the direst sort would overwhelm her and others if she ate anything—is to open the mouth with a spoon, and then, breaking across an egg, lightly boiled, and still warm, to throw its contents well back into the throat, the nose being held in the meanwhile. By this means a supply of good nutriment may be administered as often as required; whilst a repetition of it—which would be resisted successfully only in exceptional cases, a few times would generally suffice to overcome the opposition of the patient to taking nourishment; for it is well known that when such a patient recognises the ability of the physician to surmount his resistance to food, he usually gives in before this power has been many times displayed.

Besides the physician and assistant-physician, there is also an apothecary to dispense the medicines, assist in keeping the registers, and in other minor details of management. There is besides a resident chaplain, who performs daily religious service, and ministers to the sick and dying. The wards, moreover, are open to clinical pupils, and instruction by lectures on the pathology and treatment of insanity are regularly given.

The number of patients at the time of my visit was 423; of which the males were the more numerous. Dr. Verdona counted only 5 epileptic males and the same number of females, and only 4 paralytics, 2 of whom had been recently admitted. Pellagra, so rife in Lombardy, is here, as at Turin, rare: one case had recently died. In the decade between 1828 and 1838, 1738 individuals were admitted into the asylum, and the mortality stood at 37 per cent.

Criminal lunatics are received, and are found very embarrassing inmates among the rest of the population.

On a review of the impressions received on my visit to this asylum of Genoa, I must confess them to be in many respects unfavourable. The *tout ensemble* of the building on approaching it, is in aspect that of a large prison: the radiating or panoptical system of construction is, to my mind, fraught with disadvantages and discomforts. Ventilation is rendered more imperfect; the view from any portion is impeded by the neighbouring wings, and is limited and cheerless; the airing-courts are bad in figure, most of them always more or less completely in the shade, and, unless the wings are much extended in length, and at the same time of few stories, are too small to furnish exercising space for their inmates; whilst the rooms situated on each side the apex of the triangle of the divergent wings, are too near to be well lighted and ventilated. These objections need not be here extended; for they are generally recognised, and few advocates of this figure of building are to be found. The central corridor, with rooms opening on each side, is another objectionable feature in the structure of the Genoa Asylum. Much may be justly urged against a third story to an asylum, but the fourth existing in this one at Genoa, cannot be too severely condemned; it is not bad only from its elevation, but from its most faulty structure and arrangement; its position immediately beneath the roof, and within its span; its consequent shelving sides, and skylight windows; the deficient amount of air, the heat which must prevail during summer, the disposition of the dormitories whereby one is the passage to another; these defects and others, which a little reflection would suggest, are such as should lead to the abandonment of this story.

It would have been much more gratifying to have reported the readiness of the chief physician to fairly and thoroughly try the more gentle and humane system of treatment practised in Great Britain and America, and to a great extent in France and some of the best managed German asylums; but unfortunately Dr. Verdona appears to have a certain amount of prejudice against it, and consequently cannot justly appreciate its objects and advantages. That personal coercion can be so largely laid aside as reported, seems a circumstance of such doubtful complexion, that he cannot rid himself of the suspicion of a trick, nor cease to believe that it must be resorted to under another name. He has tried a padded room for a short time, in some fashion, and has been discouraged by the results obtained; and he has got the notion that the madmen he would variously fasten with straps and belts are, in non-restraint asylums, condemned to solitary confinement within a padded cell for an almost unlimited period, and as a result of the isolation are made worse. Entertaining the suspicions and fears heretofore held concerning the insane state, he has a horror of the two sexes encountering each other, and can imagine nought but ill consequences to both when they do. His opinions and practice in the treatment of insanity are in a curious hybrid state; he is just in the same position as the last race of superintendents of English asylums, when the changes at Lincoln and Hanwell were the talk of the day; every one then had such heaps of experience and practical illustrations to prove Conolly and his colleagues rash innovators,—theorists, or even (humbugs), gulling the public with their tales of homicidal maniacs at large, eating their dinners with knives, and handling edge-tools in their trades; every one knew there must be something behind the scenes; that it was all very well if half-a-dozen attendants could be put in charge of one patient, and that the cost of the destructive propensities of the patients fortunately came out of the rate-payers' pockets; or that at least no really genuine cases of violence, such as they had to manage, fell into the hands of the innovators. But these, and a score of other difficulties, doubts, and scepticisms vanished as opportunities occurred for the objectors to view matters for themselves, and to watch the development of the plan in one and another asylum. And in like manner, I doubt not, M. Verdona would lose his suspicions and let go his objections, could he see the non-restraint system in regular operation, and put himself *au courant* with the experience of British and American alienist physicians.

In conclusion, it must be a matter of regret that the Asylum of Genoa is of so recent foundation, the cost of its construction for a comparatively poor state so large, the pains taken in selecting and working out its plan so great, and the national pride in the building so considerable, that there can be no hope of seeing it replaced by another better placed in the country, and better constructed for the purposes it has to fulfil.

Unfortunately I could not obtain any statistical record of the Genoese Asylum; to complete my present paper I will therefore record a few remarks on the prevalence of insanity in Piedmont. In this small kingdom there are but four asylums in existence—those at Turin and Genoa, described, one at Alessandria, and one near Chambery. That at Alessandria I did not see, but understood that it was very indifferent as a building, small, and to be replaced by a new one. The asylum near Chambery, in Savoy, was of an equally indifferent character, being an old monastery, adapted as well as might be to its novel purpose. It contained but few patients, and a new asylum was building in the neighbourhood, under the superintendence of Dr. Duclos, to more adequately meet the wants of the province. In the large island of Sardinia no provision whatever existed for the insane, who were either the objects of fear and superstition, or the victims of harshness and cruelty, shut up in prisons, dispersed in monasteries, in hospitals, and in hospices for the vagrant poor; but alike everywhere the subjects of neglect and ill-treatment.

The proportion of insane cases, known in the asylums and elsewhere in Piedmont, as calculated by Dr. Bonacossa, is one in about every 2000 of the population; and in Savoy, according to M. Duclos, 1 in 1700. This proportion in each of these two provinces is small, more remarkably so in that of Piedmont. Between 1828 and 1838, 1738 patients were admitted into the provincial asylum of Genoa, from a population of about 580,000; whilst in the asylums of Turin and Alessandria together only 1948 were admitted, although the population of the provinces from which they receive patients is four-fifths larger than that of the Genoese territory. This circumstance M. Bonacossa believes to be explicable, in a great measure, from the difficulty of obtaining admission into the Piedmontese institutions, on account of their relative smallness, and their utter inadequacy to supply the requirements of the insane population of the extended territory for which they are the appointed receptacles. In the several provinces of Piedmont proper, *i. e.*, excluding Savoy, Genoa, and Nice, M. Bonacossa takes the number 1440 to represent the insane population requiring accommodation in asylums, and to meet this requirement proposes four new institutions, each capable of holding 300 patients; viz., one for the division of Turin, a second for that of Alessandria, a third for Novara, and a fourth for that of Cuneo. One is very much required for the province of Nice, the present plan of sending patients thence all the way to Turin, being open to the most serious objections, whether the well-being of the afflicted themselves, or the cost to the public be considered. In Savoy, as already noted, the necessity for a proper asylum has been admitted, and is by this time fully met.

M. Bini furnishes some additions to the foregoing account of the lunatic population of Piedmont, in his valuable statistical report (Saggio di Statistica del R. Manicomio di Firenze, 1854). He tells us that the careful inquiries of a Royal Commission, nominated by the King of Sardinia in 1845, show that in Piedmont alone, in a population of 2,651,106, there existed 7084 Cretins, or 2·67 to every 1000 inhabitants. If, now, he remarks, the number of the insane be added to that of the Cretins, the proportion of persons of unsound mind in Piedmont will exceed that met with in Norway and Scotland, where statistics indicate a prevalence of mental disease beyond that in other European countries.

In my next communication I shall be able to present the provisions of the law introduced into the Sardinian Parliament in 1849, for the care and treatment of lunatics.

